# Evolution and transition of expression trajectory during human brain development

**DOI:** 10.1186/s12862-020-01633-4

**Published:** 2020-06-23

**Authors:** Ming-Li Li, Hui Tang, Yong Shao, Ming-Shan Wang, Hai-Bo Xu, Sheng Wang, David M. Irwin, Adeniyi C. Adeola, Tao Zeng, Luonan Chen, Yan Li, Dong-Dong Wu

**Affiliations:** 1 State Key Laboratory of Genetic Resources and Evolution, Kunming Institute of Zoology, Chinese Academy of Sciences, Kunming, 650223 Yunnan China; 2 Kunming College of Life Science, University of the Chinese Academy of Sciences, Kunming, 650223 Yunnan China; 3 State Key Laboratory of Cell Biology, Shanghai Institute of Biochemistry and Cell Biology, Center for Excellence in Molecular Cell Science, Chinese Academy of Sciences, University of Chinese Academy of Sciences, Shanghai, 200031 China; 4 Department of Laboratory Medicine and Pathobiology, University of Toronto, Toronto, Ontario M5S 1A8 Canada; 5 Banting and Best Diabetes Centre, University of Toronto, Toronto, Ontario M5G 2C4 Canada; 6 Key Laboratory of Systems Biology, Hangzhou Institute for Advanced Study, University of Chinese Academy of Sciences, Chinese Academy of Sciences, Hangzhou, 310024 China; 7 Center for Excellence in Animal Evolution and Genetics, Chinese Academy of Sciences, Kunming, 650223 Yunnan China; 8 State Key Laboratory for Conservation and Utilization of Bio-Resource, Yunnan University, Kunming, 650091 Yunnan China

**Keywords:** Humans, Macaques, Expression trajectory, Transcriptome, Brain evolution

## Abstract

**Background:**

The remarkable abilities of the human brain are distinctive features that set us apart from other animals. However, our understanding of how the brain has changed in the human lineage remains incomplete, but is essential for understanding cognition, behavior, and brain disorders in humans. Here, we compared the expression trajectory in brain development between humans and rhesus macaques (*Macaca mulatta*) to explore their divergent transcriptome profiles.

**Results:**

Results showed that brain development could be divided into two stages, with a demarcation date in a range between 25 and 26 postconception weeks (PCW) for humans and 17-23PCWfor rhesus macaques, rather than birth time that have been widely used as a uniform demarcation time of neurodevelopment across species. Dynamic network biomarker (DNB) analysis revealed that the two demarcation dates were transition phases during brain development, after which the brain transcriptome profiles underwent critical transitions characterized by highly fluctuating DNB molecules. We also found that changes between early and later brain developmental stages (as defined by the demarcation points) were substantially greater in the human brain than in the macaque brain. To explore the molecular mechanism underlying prolonged timing during early human brain development, we carried out expression heterochrony tests. Results demonstrated that compared to macaques, more heterochronic genes exhibited neoteny during early human brain development, consistent with the delayed demarcation time in the human lineage, and proving that neoteny in human brain development could be traced to the prenatal period. We further constructed transcriptional networks to explore the profile of early human brain development and identified the hub gene *RBFOX1* as playing an important role in regulating early brain development. We also found *RBFOX1* evolved rapidly in its non-coding regions, indicating that this gene played an important role in human brain evolution. Our findings provide evidence that *RBFOX1* is a likely key hub gene in early human brain development and evolution.

**Conclusions:**

By comparing gene expression profiles between humans and macaques, we found divergent expression trajectories between the two species, which deepens our understanding of the evolution of the human brain.

## Background

Our highly developed and distinctive brains, which set humans apart from other mammals, are the product of evolution [1, 2], the mechanism of which has fascinated people for centuries [3]. Based on compelling differences in cognitive and behavioral capacities, but relatively close phylogenetic relationship between humans and non-human primates (NHPs) [4–6], recent comparative analyses have provided a novel strategy to study human-specific neurodevelopment [7–9]. Increasingly persuasive evidence suggests that brain development is not static but is a continuous process of molecular changes throughout life, including changes in gene expression, glucose metabolism, and synaptic density [10–14]. Previous comparative analyses between humans and NHPs have only offered a snapshot in time [15, 16]. However, it is necessary to compare the whole process of brain development to provide a more objective and comprehensive understanding of human brain evolution.

Earlier research noted that neurodevelopmental timing is impacted by different developmental rates and life history strategies [2]. These differences in neurodevelopmental timing among species, also called heterochrony, have long been considered a crucial impetus for evolution [17–19]. Humans have an unusually extended childhood and slow rate of neurodevelopment (known as neoteny) relative to other animals, which is considered a possible mechanism for human brain evolution [18, 20]. While previous studies have primarily focused on heterochronic gene expression during postnatal brain development [20], the macroscopic layout of the brain is nearly complete at the time of birth [21]. Thus, extending comparative analysis to the prenatal stages is necessary for exploring the features of neurodevelopment.

Currently, it is widely accepted that changes in spatiotemporal gene expression play a critical role in the emergence of the sophisticated human brain, and several attempts have been made to estimate divergent gene expression patterns between humans and NHPs [15, 22, 23]. However, our understanding of how gene expression patterns have changed in the human lineage remains incomplete. With increasing high-quality brain transcriptome data [24–26], an unprecedented opportunity to investigate gene expression trajectory in multiple brain regions and different developmental stages among primates has become possible [22, 27]. In this study, we collected large-scale gene expression data from humans and macaques to systematically investigate and compare their divergent gene expression trajectory. We aimed to identify critical states during brain development as well as novel molecular mechanisms underlying human brain evolution.

## Results

### Study design

**Fig. 1:**
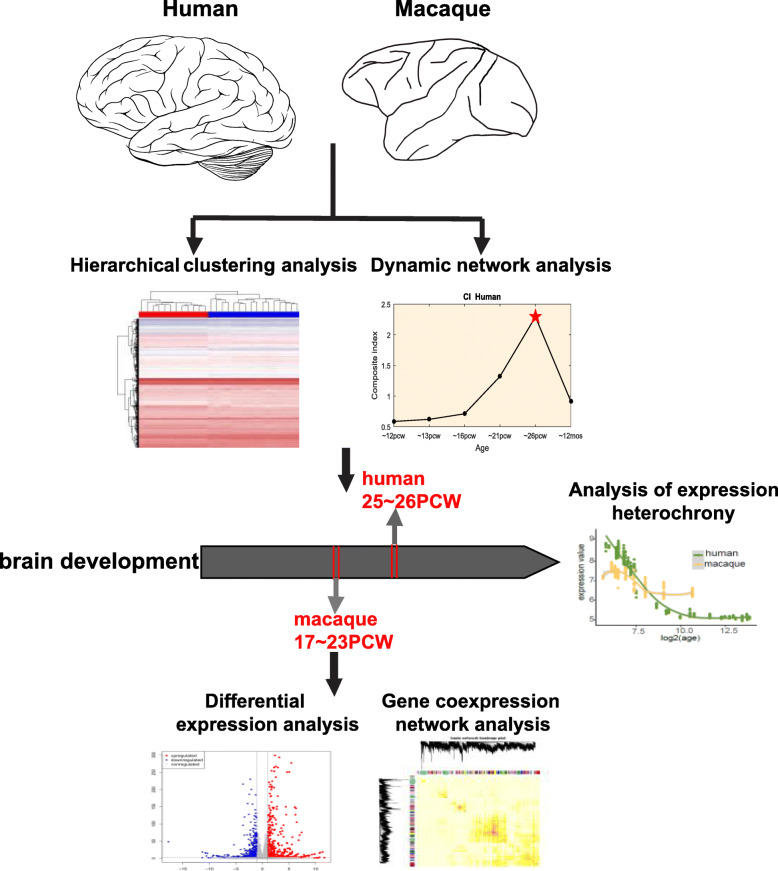
Overview of study. Hierarchical clustering and dynamic network analyses were used to identify demarcation time of brain development in humans and macaques. Expression heterochrony analysis was used for exploring the mechanism of neurodevelopmental timing between humans and macaques. Differential expression and gene co-expression network analyses were used to identify key genes in human brain development and evolution. The clipart depicted in the figure are original

Figure 1 highlights the strategy used to investigate evolution of gene expression trajectory in humans, including hierarchical clustering and dynamic network analyses to identify demarcation times of brain development in humans and macaques, expression heterochrony analysis to explore the mechanism of neurodevelopmental timing in humans, and differential expression and gene co-expression network analyses to identify key genes in human brain development and evolution.

Human brain transcriptome data were used in this study, including RNA-sequencing (RNA-seq) and microarray data across multiple brain regions downloaded from the Allen Brain Atlas [24, 25] (Table 1; Additional file 1: Table S1 ~ Table S2). These data cover 14 brain regions spanning 27 different developmental ages from 8 postconception weeks (*PCW*) to postnatal 40 years old (Table 2). In total, 30,881 and 17,280 genes had detectable expression signals in the RNA-seq and microarray data, respectively. We also used microarray data from macaques, which contained five brain regions (22 brain subregions) corresponding to brain regions in humans (Table 1; Additional file 1: Table S3 ~ Table S4) and spanning 8 PCW to postnatal 48 months (Table 2). In total, 15,381 genes exhibited detectable expression signals.

**Table 1:** Human and macaque tissues used in this study

**Human**	A1C	Primary auditory cortex
	**AMY**	Amygdaloid complex
	MD	Mediodorsal nucleus of thalamus
	DFC	Dorsolateral prefrontal cortex
	**HIP**	Hippocampus
	IPC	Posteroventral (inferior) parietal cortex
	ITC	Inferolateral temporal cortex
	M1C	Primary motor cortex
	**ACC**	Anterior (rostral) cingulate cortex
	OFC	Orbital frontal cortex
	STC	Posterior (caudal) superior temporal cortex
	**STR**	Striatum
	**V1C**	Primary visual cortex
	VFC	Ventrolateral prefrontal cortex
**Macaque**	HIP	CA1or (stratum oriens of CA1)
		CA1py (stratum pyramidale of CA1)
		CA1ra (stratum radiatum of CA1)
		CA2py (stratum pyramidale of CA2)
		CA3py (stratum pyramidale of CA3)
		DGgr (granular layer of dentate gyrus)
		DGpf (polyform layer of dentate gyrus)
		DGsg (subgranular zone of dentate gyrus)
	STR	NAC (nucleus accumbens)
		ic (internal capsule)
		Pu (putamen)
		(Gpe) External segment of globus pallidus
		Internal segment of globus pallidus
	ACC	(rCG2) Layer II of rostral cingulate cortex
		(rCG3) Layer III of rostral cingulate cortex
		(rCG5) Layer V of rostral cingulate cortex
		(rCG6) Layer VI of rostral cingulate cortex
	V1C	(V1–3) Layer III of V1
		(V1–4) Layer IVA of V1
		(V1–5) Layer V of V1
	AMY	(Me) Medial nucleus of amygdaloid
		(PL) Paralaminar nucleus

The brain regions marked with bold in human represent that coexist with macaque

**Table 2:** Age distribution of humans and macaques

Human		Macaque	
Early period	Later period	Early period	Later period
56 PCD (8 PCW)	182 PCD (26 PCW)	50 PCD (8 PCW)	0 m
72 PCD (9 PCW)	4 m	70 PCD (10 PCW)	3 m
84 PCD (12 PCW)	10 m	80 PCD (12 PCW)	12 m
91 PCD (13 PCW)	1 y	90 PCD (13 PCW)	48 m
112 PCD (16 PCW)	2 y	120 PCD (17 PCW)	
119 PCD (17 PCW)	3 y		
133 PCD (19 PCW)	4 y		
147 PCD (21 PCW)	8 y		
168 PCD (24 PCW)	13 y		
175 PCD (25 PCW)	15 y		
	18 y		
	21 y		
	23 y		
	30 y		
	36 y		
	37 y		
	40 y		

*PCW* postconception week, *PCD* postconception days, *m* month, *y* year

### Different developmental trajectories and demarcation times in humans and macaques

**Fig. 2:**
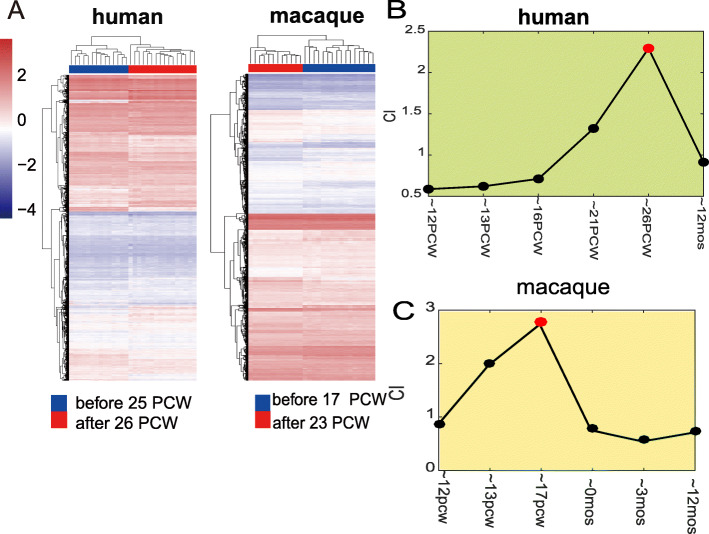
Different gene expression trajectories during brain development in humans and rhesus macaques. **a** Hierarchical clustering analysis revealed different expression demarcation time points in humans and macaques based on primary visual cortex transcriptome data. **b** Time course for neurogenesis in humans and macaques. Data were according to previous study [10]. **c–d** Detection of transition phases during brain development in humans (**c**) and macaques (**d**) using dynamic network biomarkers (DNBs). Plot represents composite index of DNB (see Materials and methods, CI in Eq.(1)), which indicates transition phase at around 26 PCW in humans and around 17 PCW in macaques

We performed hierarchical clustering analysis based on gene expression levels to determine whether transformation exists during brain development. In humans, the RNA-seq and microarray data supported the division of brain development into two stages, with a demarcation time of 25–26 PCW (Fig. 2a; Additional file 2: Figure S1). In macaques, gene expression levels from the microarray data in most brain regions were also clustered into two groups, with a demarcation time of 17 PCW ~ birth (17 -23PCW)(Fig. 2a; Additional file 2: Figure S2). These results suggest that brain development in both humans and macaques could be divided into two major stages, separated by species-specific demarcation points that occurred prior to birth (25–26 PCW in humans and 17–23 PCW in macaques), rather than birth times, which have been widely used as a uniform demarcation time of neurodevelopment across species [28, 29].

### Transitional state and critical transitions during brain development based on dynamic network biomarker (DNB) analysis

Having identified the demarcation points of brain development in humans and macaques, we further applied DNB analysis to verify whether the above demarcation points were also transitional states of brain development. Based on nonlinear dynamic theory, biological processes, such as brain development, are not always smooth, but can exhibit dramatic transitions from one state to another [30, 31]. When the process occurs near a critical transition phase, a dominant group of genes/molecules, i.e., DNBs, can drive transition of the dynamic process [30, 32, 33]. We thus performed a genome-wide DNB analysis to identify the transitional states and DNB genes in both humans and macaques.

The DNB results demonstrated that the transitional states of brain development in humans and macaques occurred at around 26 PCW and around 17PCW, respectively (Fig. 2b-c; Additional file 2: Figure S3). After the transitional state, gene expression patterns changed markedly. The transitional states identified by DNB analysis were largely consistent with the demarcation points above (Fig. 2a), thus supporting the robustness of our results. We also obtained the corresponding DNBs, which included 369 DNB genes in humans and 34 DNB genes in macaques (Additional file 1: Table S5). The greater number of DNB genes in humans suggests more dramatic changes between the early and later stages of brain development in humans relative to that in macaques.

### Transcriptional profile and cell fate change from early to later stages of brain development

**Fig. 3:**
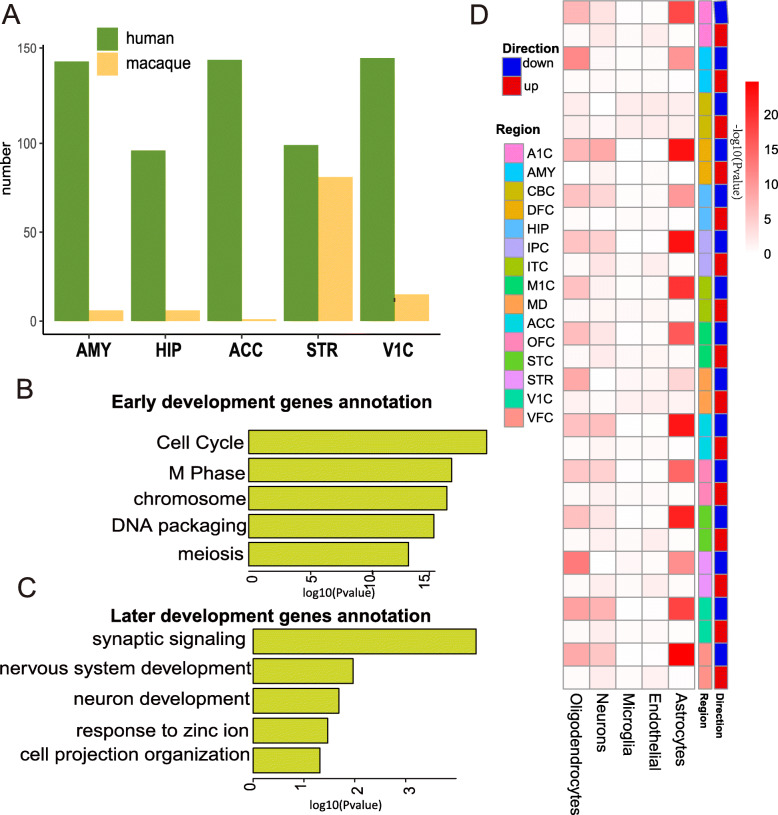
Transcriptional profiles across early to later stages during brain development. **a** DEGs among five brain regions (HIP (hippocampus), V1C (primary visual cortex), ACC (anterior cingulate cortex), STR (striatum), and AMY (amygdala). **b** Enriched categories for up-regulated genes in early human brain development. **c** Enriched categories for up-regulated genes in later human brain development. **d** Matrix summary of enrichment of oligodendrocyte, neuron, microglia, endothelial, or astrocyte genes [34] in DEGs up-regulated (red) and down-regulated (blue) in each human tissue

We used differential expression analysis to compare the degree of change in early and later gene expression between humans and macaques. We selected five brain regions (i.e., hippocampus (HIP), striatum (STR), anterior cingulate cortex (ACC), amygdala (AMY), and primary visual cortex (V1C)) that coexist and contain similar sample sizes in the two species. Based on microarray probes, which matched between humans and macaques, we found a larger number of differentially expressed genes (DEGs) (Benjamini-Hochberg FDR < 0.05, fold change [FC] > 1.5) between early and later stages in humans than in macaques (Fig. 3a; Additional file 1: Table S6). These results also suggest more dramatic changes between early and later stages of brain development in humans relative to macaques.

To better understand brain developmental processes in the human lineage, we conducted functional enrichment analysis of the DEGs between early and later stages in humans. Results showed that up-regulated genes in the early stage were mainly involved in cell cycle, DNA packaging, and meiosis (Fig. 3b), whereas, up-regulated genes in the later stage were enriched in synaptic signaling, myelination, and axon establishment (Fig. 3c). Remarkably, the DEG patterns well matched the reported properties of the neurodevelopmental timeline in humans [35].

Increasing evidence suggests that a cell fate switch from neurons to glial cells is operational in prenatal brains and represents a key process in brain development [36–38]. We thus considered whether the demarcation points in human correspond to the transient time of known neuron to glial cell fate switch. We found up-regulated genes in the early stage were predominantly enriched in neuronal genes (Fig. 3d), whereas up-regulated genes in the later stage represented a diversity of cell-type-associated genes, including astrocytes, oligodendrocytes, and neurons (Fig. 3d), which likely reflects the transformation of cell fate switch from neurons to glial cells during the demarcation point. This conclusion is confirmed by the previous single-cell transcription analysis [39], which reported that neurons developed from neural progenitor cells in early gestational weeks (GW8, GW9, GW10, GW12, GW13, GW16, GW19, GW23), whereas glial cells (oligodendrocyte progenitor cells and astrocytes) differentiated from neural progenitor cells in later weeks (GW26).

Earlier studies have also reported several pathways that govern the neuron to astrocyte cell switch, including the gp130/JAK/STAT and MEK/MAPK pathways … [38, 40]. We found that DEGs between early and later stages were significantly enriched in the MEK/MAPK pathway (*P* = 4.6e-04, Fisher’s exact test) (Additional file 1: Table S7). Although DNBs were not significantly enriched in the MEK/MAPK pathway (*P* = 0.15, Fisher’s exact test), 15 DNBs were still involved (Additional file 1: Table S7). This suggests that the MEK/MAPK pathway likely plays an important role in the transformation ratio of cell type from neurons to glial cells during the human demarcation point (25–26 PCW).

### Molecular mechanism underlying protracted timing of early human brain development

The different demarcation points (25–26 PCW in humans and 17-23PCWin macaques) identified here reflect prolonged timing of early brain development in the human lineage. Thus, we performed heterochronic analysis to explore the molecular mechanism underlying different timing of early neurodevelopment between humans and macaques.

**Fig. 4:**
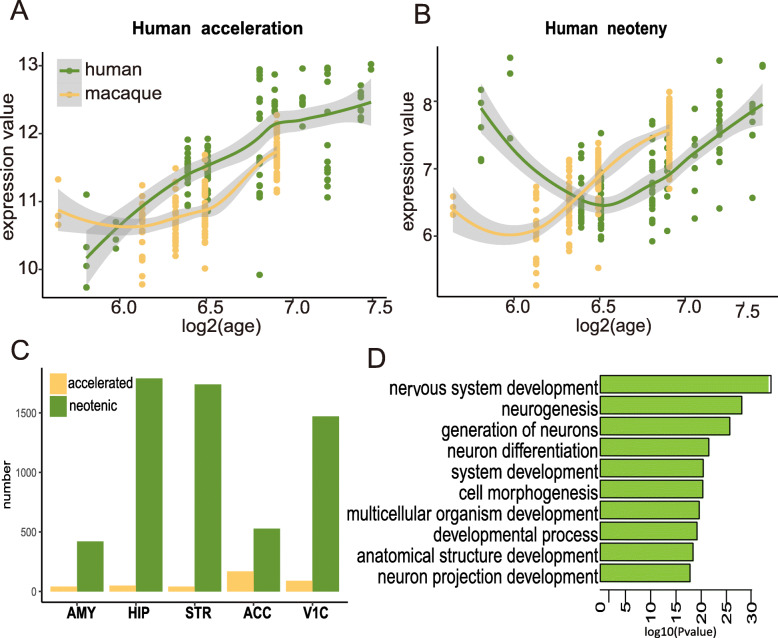
Analysis of expression heterochrony. **a** Example gene showing accelerated expression in humans. **b** Example gene showing neotenic expression in humans (right). **c** Number of genes showing acceleration and neoteny in early human brain development for five brain regions. **d** Enriched categories for neotenic genes in early human brain development

We again used five brain regions (i.e., HIP, STR, ACC, AMY, and V1C) that coexist in humans and macaques to test heterochronic gene expression. After rigorous quality control (see Methods), we retained 9758 genes with microarray probes well matched between humans and macaques. Among these genes, we selected several that showed both age-related and species-specific differences for each brain region (see Methods; Additional file 1: Table S8). We then sorted these genes into two categories: (*i*) human acceleration genes, whose expression change was significantly faster during human brain development than that during macaque brain development (Fig. 4a); and, (*ii*) human neoteny genes, whose expression change was significantly delayed during human brain development than that during macaque brain development (Fig. 4b), as defined in previous study [20]. Compared to macaques, more genes displayed a neotenic pattern in all five human brain regions (Fig. 4c; Additional file 1: Table 9), consistent with the above delayed demarcation point in the human lineage. In addition, the result suggest that neoteny of human brain development could be traced to prenatal period.

Interestingly, the neotenic genes from the five brain regions significantly overlapped (Additional file 1: Table 10), suggesting that neotenic mechanisms among different brain regions are largely convergent. The functions of these neotenic genes were mainly involved in neurodevelopment-related pathways (Fig. 4d), suggesting that more neurodevelopmental genes exhibited neotenic features, which eventually help humans develop a more complex brain.

### Co-expression analysis identifies gene network during early brain development in humans

**Fig. 5:**
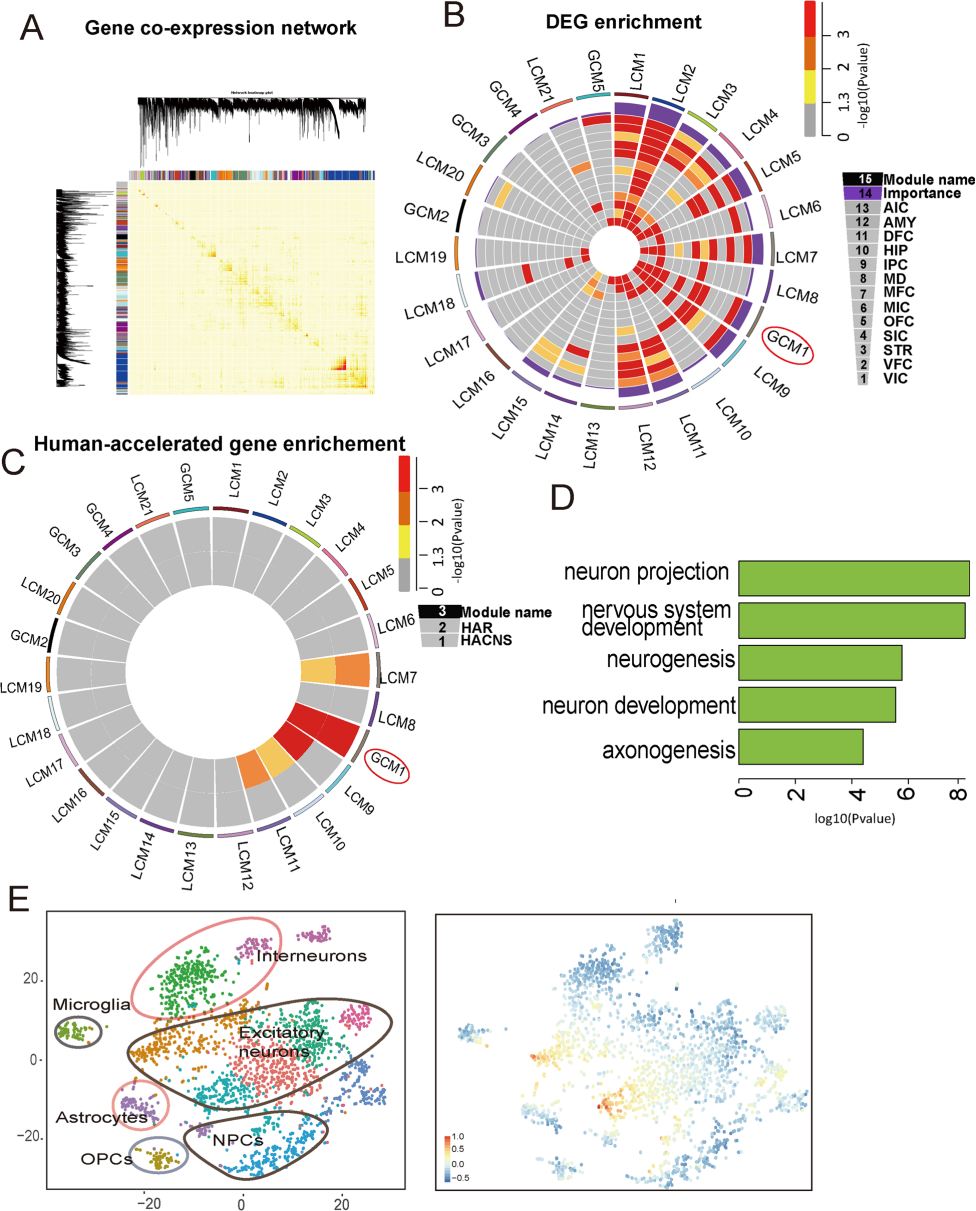
Weighted gene co-expression network analysis (WGCNA). **a** Topological overlap matrix plots for early brain modules in human. Light color represents low topological overlap, with progressively darker red representing higher overlap. **b** Enrichment of DEGs across 13 brain regions among different modules. **c** Enrichment of genes located in human-accelerated conserved non-coding sequences (HACNSs) [44] and genes located in human DNA sequence accelerated regions (HARs) [45] in different modules. **d** Functional enrichment of genes in GCM1. **e** Cell specificity of genes in module GCM1

Extended timing of early neurodevelopment in humans is important for brain evolution [2, 18]. We applied weighted gene co-expression network analysis (WGCNA) to further explore the transcriptional profile of early neurodevelopment in humans [41–43]. A total of 38 modules related to early human neurodevelopment were identified (see Methods; Fig. 5a; Additional file 1: Table S11). To quantify network reorganization across early and later brain development, we applied modular differential connectivity (MDC), which is the ratio of the average connectivity for any pair of modules sharing genes in the early stage compared to that in the later stage. Among the 38 early modules, five (GCM1–GCM5) showed gain of connectivity compared to later development, with co-regulation enhanced between genes in these modules. In contrast, 21 modules (LCM1–LCM21) showed loss of connectivity and 12 modules (NCM1–NCM12) (31.5%) showed no change in connectivity and were conserved during development (Additional file 2: Figure S4A-S4B).

For modules with gains or losses of connectivity, we ranked them according to their degree of DEG enrichment across brain regions (Fig. 5a) and MDC scores (Additional file 1: Table S11). Module GCM1, which showed a gain of connectivity in the early stage, was identified as the most highly ranked module. The genes in this module were enriched in neurogenesis, neuron projection morphogenesis, and axon development (Fig. 5d). Additionally, GCM1 showed highly significant enrichment for known autism susceptibility markers (*P* = 5.49E-07; Fisher’s exact test) [46], and the expression levels of genes in this module were significantly higher during early brain development (Additional file 2: Figure S4C). These results suggest that GCM1 likely plays an important role in early development of the human brain.

Remarkably, most genes in the GCM1 module were located in human-accelerated conserved non-coding sequences (HACNSs) (*P* = 3.19E-16; Fisher’s exact test) [44] or in human DNA sequence accelerated regions (HARs) (*P* = 7.49E-12; Fisher’s exact test) [45] (Fig. 5c), suggesting that genes in the GCM1 module also likely played an important role in human brain evolution.

We next mapped the genes in GCM1 to single-cell expression data derived from 20,262 prenatal human prefrontal cortex cells that ranged in age from 8 to 26 gestational weeks [39] (Additional file 2: Figure S5), which represent a broad diversity of cell types including neural progenitor cells, interneurons, astrocytes, oligodendrocyte progenitor cell, microglia and excitatory neurons. The expression patterns of the GCM1 genes closely matched the cell type specific of excitatory neurons (see Methods) (Fig. 5e), confirming that genes in the GCM1 module function through excitatory neurons.

**Fig. 6:**
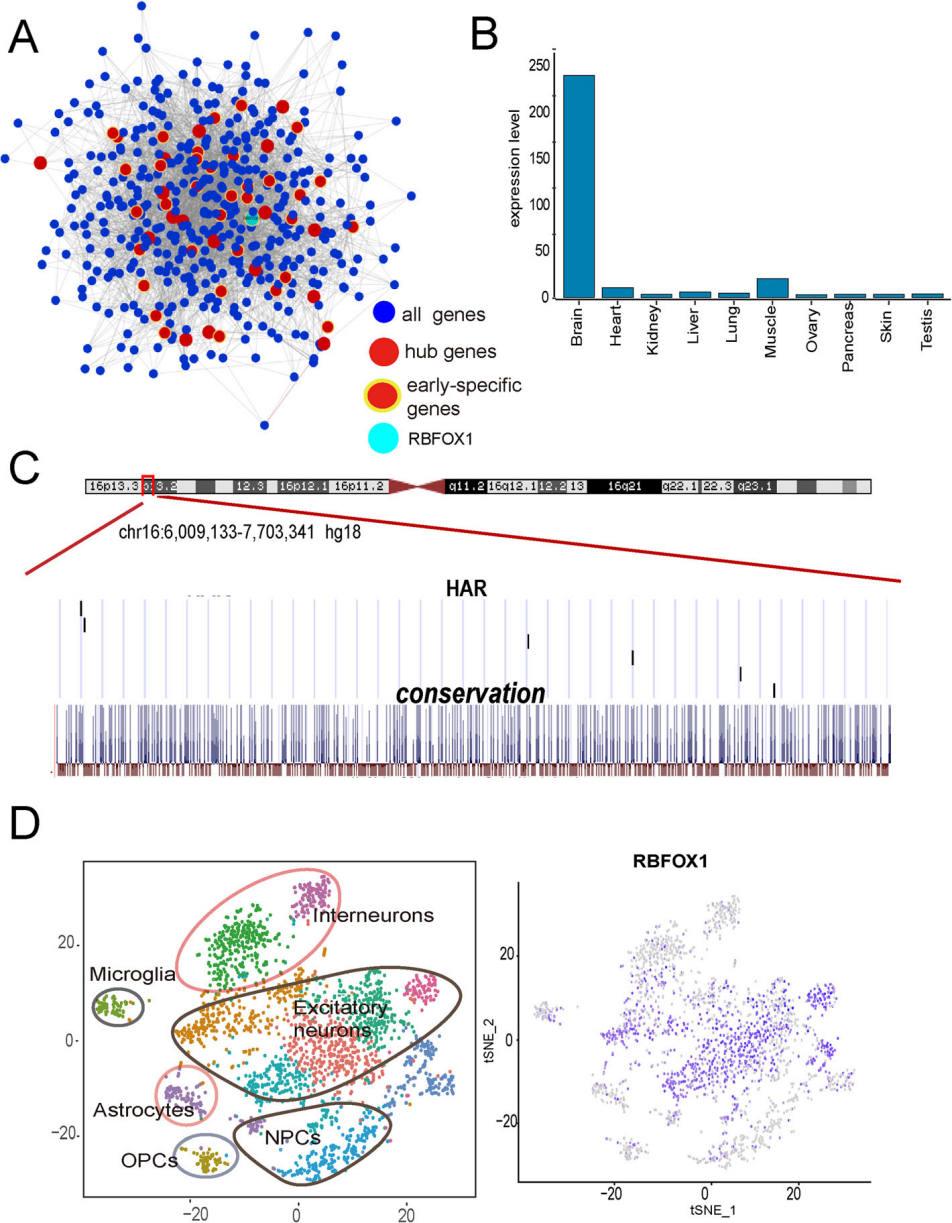
Hub gene *RBFOX1* in module GCM1. **a** Network plot of hub genes identified within GCM1 module. Blue nodes indicate all genes. Red nodes indicate hub genes. Yellow halos indicate early stage-specific hub genes. Cyan node indicates *RBFOX1*. Edges reflect significant interactions between genes based on mutual information. **b** Expression level of *RBFOX1* in different tissues. **c** Location of HAEs at *RBFOX1* locus in human genome and conservation of *RBFOX1* among 17 mammals according to UCSC Genome Browser (www.genome.ucsc.edu). **d** Cell specificity of *RBFOX1*

We further reconstructed the network structure of genes within the GCM1 module based on their connectivity and identified 53 hub genes, 36 of which were early stage-specific hub genes [47] (see Methods, Fig. 6a). Among these genes, *RBFOX1*(RNA Binding Fox-1 Homolog 1) was of particular interest. *RBFOX1* is a highly conserved splicing regulator that displays higher expression in the brain than in other tissues [48] (Fig. 6b). *RBFOX1* is implicated in autism, epilepsy syndromes, and Alzheimer’s disease [49–51] and plays an important role in mammalian brain development [52]. Interestingly, *RBFOX1* is also a DNB gene, and therefore considered to play an important role in critical transition during brain development [30]*.*

We next used evolutionary analysis to test if *RBFOX1* experienced positive selection in the human lineage. Although the protein coding sequence of *RBFOX1* has not changed in humans compared to other primates (Additional file 2: Table S12; see Methods), six HARs were found in the non-coding regions for this gene (Fig. 6c). HARs are non-coding regions conserved across mammals, and which have acquired many sequence changes in humans since their divergence from chimpanzees [45]. Only seven human RefSeq genes from the entire human genome (based on hg18) contain six or more HARs [53], suggesting that strongly human-specific accelerated evolution occurred recently in the non-coding region of the human *RBFOX1* gene. Our findings provide evidence that *RBFOX1* is a likely key hub gene in early human brain development and evolution. In addition, *RBFOX1* also showed cell specificity to excitatory neurons by single cell transcriptome analysis (Fig. 6d), suggesting that *RBFOX1* functions through excitatory neurons.

## Discussion

The remarkable abilities of the human brain set us apart from NHPs. With the advent of large-scale genomic, transcriptomic, and epigenomic data, many genetic underpinnings of the rapid evolution of the human brain have been revealed [54–58]. However, our understanding of how the brain has changed in the human lineage remains incomplete [3]. Based on large-scale transcriptomic and genomic data, the results of the current study provide new insight into the evolution and transition of gene expression trajectory in the human brain.

Firstly, we found that brain development could be divided into two stages in both humans and macaques; more specifically, demarcation times of 25–26 PCW and 17–23 PCW in humans and rhesus macaques, respectively. Further DNB analysis indicated that the demarcation points were nearly the same as the critical transitional states during brain development in humans and macaques. Previous studies on brain development have primarily used birth as the default boundary [28, 29, 59]. However, we suggest that the demarcation points identified here should be considered in the future to minimize biases in studies of brain development.

Secondly, we also found that neoteny of human brain development could be traced to the prenatal period. Previous studies have primarily focused on heterochronic gene expression during postnatal brain development [7]. The macroscopic layout of the brain is nearly complete at the time of birth [8]. Thus, extending comparative analysis to the prenatal stages is necessary to explore the features of neurodevelopment, which is lacking in prior studies. Thus, in this paper, we performed heterochronic analysis across prenatal samples between humans and macaques, and found that more genes displayed a neotenic pattern in humans than in macaques, consistent with the delayed demarcation time in the human lineage, and proving that neoteny in human brain development could be traced to the prenatal period.

Thirdly, we used gene co-expression network analysis to identify transcription profiles in early human brain development and identified that the *RBFOX1* gene likely plays an important role in early human brain development and displays positive selection in its non-coding region [50–52]. Therefore, we speculated that*RBFOX1* is a likely key hub gene in early human early brain development and evolution. As such, we propose that *RBFOX1* should be considered in further neurodevelopmental research.

Finally, we highlighted the importance of excitatory neurons in human brain development and evolution. Over the past few decades, the comparisons of excitatory neurons between humans and NHPs have mainly focused on their differences in morphology and abundance [60, 61]. Further molecular biology research on excitatory neurons is limited. In this paper, we found that the GCM1 module and *RBFOX1* gene were related to early human brain development and evolution and were enriched in excitatory neurons. Therefore, studies on excitatory neurons would be promising for exploring human brain evolution.

We note that the study presented here is far from being comprehensive: Firstly, Based on currently available transcriptome data, we identified a demarcation line time-frame of brain development in humans and macaques. The precise demarcation point could not be concluded from existing data but should be explored in future studies.

Secondly, due to the relatively small sample sizes used in the current study, as well as the sparse distribution of samples across ages, we cannot rule out certain important changes in transcriptional profiles during neurodevelopment that may have occurred beyond the sampling range used in this study. For instance, previous studies have reported that during juvenile development in humans (1–8 postnatal years), the cerebral cortex consumes nearly twice the amount of glucose as observed during adulthood and is accompanied by dramatic changes in synaptic density during that developmental window [11, 12]. Thus, the transition state we identified is not absolute, with more saturated samples across different ages required to confirm our conclusions.

Thirdly, comparative analysis between humans and macaques was based on microarray data only, which rely on pre-existing knowledge of RNA sequences; as such, some important genes may be missed.

Finally, due to the lack of prenatal transcriptome data on brain development in hominoids, it is difficult to compare hominoids with humans, which would be valuable when exploring human brain evolution.

Further analyses, including expression data analysis across additional development stages, comparative analysis of RNA-seq data between humans and NHPs, as well as analysis incorporating more hominoids, are needed to expand our results.

## Conclusions

In this study, we integrated transcriptomic analysis to reveal the evolution and transition of expression trajectories during human brain development. By comparing gene expression profiles between humans and rhesus macaques, our results provide new insights into the gene expression trajectory of human brain development, which will deepen our understanding on evolution of the human brain.

## Methods

### Dataset resources

The normalized gene expression human and rhesus macaque datasets were obtained from the Allen Brain Atlas (http://www.brain-map.org) (Table 1; Table 2) [24, 25]. We used two datasets for humans, including RNA-seq and microarray data, which contained 14 brain regions and 27 developmental stages. The RNA-seq data were summarized to Gencode 10 gene-level reads per kilobase million mapped reads (RPKM), whereas the microarray data were based on the Affymetrix GeneChip Human Exon 1.0 ST Array platform. Several quality control measures were implemented to reduce errors due to spatial artifacts on the chips, technical differences between chips in probe saturation, or other unaccounted for batch effects. Detailed information can be found in the Supplementary Materials of Kang et al. (2011) [24]. For rhesus macaques, we used the microarray dataset based on GeneChip Rhesus Macaque Genome Arrays from Affymetrix. From the 52,865 probe sets included in the macaque microarray data, 12,441 high-confidence probe sets were retained after quality control filtering. Detailed description of the macaque data can be found in Bakken et al. (2016) [25]. The macaque microarray dataset contained five brain regions (22 brain subregions) and nine developmental stages (Table 1; Table 2).

### Clustering of genes in each tissue

The human microarray and RNA-seq datasets and rhesus macaque microarray dataset were used to cluster genes for each brain region according to their expression levels. To reduce the influence of technical noise, only genes with expression values of more than 0 in 80% of the available samples were considered expressed. Before clustering, we log2 transformed and then z-transformed the expression levels (normalized the mean to 0 and variance to 1). Using agglomerative hierarchical clustering with the average and complete methods in the flashClust R package [62], the RNA-seq and microarray data from most human tissues were clustered into two groups, separated with a demarcation point of 25 PCW. The microarray data from the rhesus macaques were also clustered into two groups, with 17 PCW as the demarcation point.

### Dynamic network biomarker (DNB) analysis

Based on DNB theoretical analysis [30, 32, 33], we can prove that when a system is near the critical state/transition phase, a dominant group of genes/molecules, i.e., DNBs, can drive transition of the dynamic process. These molecules must satisfy the following three criteria [30]:

1. Deviation (or fluctuation) for each molecule inside the dominant group (*SD*_*d*_: standard deviation) drastically increases.
2. Correlation between molecules inside the dominant group (*PCC*_*d*_: Pearson correlation coefficients in absolute values) rapidly increases.
3. Correlation between molecules inside and outside the dominant group (*PCC*_*o*_: Pearson correlation coefficients in absolute values) rapidly decreases.

The dominant group is considered a DNB and plays an important role in phase transition. A quantification index (CI) considering all three criteria can then be used as the numerical signal of the critical state or transition phase and also for the identification of DNB members/molecule, with the following equation:

1 $$CI=: \frac{SD_d\bullet {PCC}_d}{PCC_o}$$

where, *PCC*_*d*_ is the average Pearson’s correlation coefficient (PCC) between the genes in the dominant group (or DNB) of the same time stage in absolute value; *PCC*_*o*_ is the average PCC between the dominant group (or DNB) and others of the same time stage in absolute value; and, *SD*_*d*_ is the average standard deviation (SD) of genes in the dominant group (or DNB). The three criteria together construct the composite index (CI). The CI is expected to peak or increase sharply during the measured stages when the system approaches the critical state, thus indicating imminent transition or transition phase of the biological process [30].

We applied this DNB method to detect the critical points and DNB members in humans and macaques based on the transcriptome data of the primary visual cortex. In each sampling stage, there were 1–4 samples with gene expression profiles. To increase the reliability of the DNB results, the slide window method was incorporated into the DNB model to process data [30].

### Differentially expressed genes (DEGs) between early and later stages of brain development

To remove the potential effect of different high-throughput platforms on gene expression values, we only used microarray data for DEG analysis for both species. Pairwise differential expression was investigated using the edgeR R package [63]. To determine the DEGs between the two developmental times for humans and macaques, the demarcation times were set 25 PCW and 17 PCW, respectively. A nominal significance threshold of Benjamini-Hochberg FDR < 0.05 and fold change [FC] > 1.5 was used to identify DEGs.

For DEGs in humans, we applied g:Profiler (https://biit.cs.ut.ee/gprofiler/) [64] for functional annotation analysis (GO and KEGG). To assess cell-type specificity in the 14 brain regions of humans, we used genes expressed at least five-fold higher in one cell type than all other cell types (neuron, microglia, astrocyte, oligodendrocyte, endothelial) from brain-based RNA expression data as the cell marker [65].

### Heterochrony analyses with dynamic time warping algorithm (DTW-S)

We combined data from microarray probes of humans and macaques to study heterochronic gene expression in five brain regions (i.e., hippocampus (HIP), striatum (STR), anterior cingulate cortex (ACC), amygdala (AMY) and primary visual cortex (V1C)). We used the “sva” R package [66] to remove batch effects between microarray datasets of humans and rhesus macaques.

To choose age-related genes, we first used a log2 transformed age scale to ensure a more linear relationship between age and phenotype [67]. We tested the effect of age on the expression levels using polynomial regression models, as described previously [68]. We next tested each gene for expression divergence between humans and rhesus macaques using analysis of covariance [69] (F-test *P* < 0.05). Identification of age-related genes and species-specific genes were based on the adjusted r2 criterion. The identification methods have been described previously [68]. Consequently, we selected 892 genes in AMY, 2431 genes in HIP, 1961 genes in STR, 1899 genes in ACC, and 2416 genes in V1C as the test gene set for DTW-S, satisfying the following criteria: (i) significant expression change with age and (ii) significant expression difference between humans and macaques.

The DTW-S algorithm was then used to analyze the data for heterochrony [68]. We defined genes showing significant heterochrony into two categories: (*i*) human acceleration genes, whose expression changes were significantly faster during human brain development than that during macaque brain development; and, (*ii*) human neoteny genes, whose expression changes were significantly delayed during human brain development compared with that during macaque brain development. Using those genes that showed significant age-related and species-specific differences, as defined above, we aligned the macaque and human expression trajectories and estimated the time-shift (heterochrony) between humans and macaques, with simulations conducted to estimate the significance of the shifts. We considered genes as ‘significantly heterochronic’ if they showed a shift at *P* < 0.05. A detailed description of the DTW-S algorithm can be found in Yuan et al. 2011 [68].

### Construction of gene co-expression modules for early human brain development

We constructed multi-tissue co-expression networks that simultaneously captured intra- and inter-tissue gene-gene interactions using the human RNA-seq expression data [42, 70]. Before identifying co-expressed gene modules, we used linear regression to correct sex and brain region covariates in the expression data. To quantify the differences in the transcript network organization between the early and late stages, we employed the modular differential connectivity (MDC) metric [71]. In brief, MDC represents the connectivity ratios of all gene pairs in a module from the early stage to the same gene pairs from the later stage: MDC > 0 indicates a gain of connectivity or enhanced co-regulation between genes in the early stage, whereas MDC < 0 indicates a loss of connectivity or reduced co-regulation between genes in the early stage.

To identify key regulator (driver) genes of the GCM1 module, we applied key driver analysis to the module-based unweighted co-expression networks derived from ARACNE [47]. ARACNE first identified significant interactions between genes in the module based on their mutual information and then removed indirect interactions through data processing inequality (DPI). For each ARACNE-derived unweighted network, we further identified the key regulators by examining the number of *N*-hop neighborhood nodes for each gene.

### Identification of cell type and subtype from single cell data

Single-cell RNA-seq data (accession number GSE104276) were reported in previous study [39]. Transcript counts for each cell were normalized to transcripts per million (TPM), with the TPM values then normalized by log ((TPM/10) + 1) for subsequent analysis [39]. The Seurat package [72] v1.2.1 implemented in R was applied to identify major cell types among the 2394 single cells from the prefrontal cortex. Only cells that expressed more than 1000 genes were considered, and only genes with normalized expression levels greater than 1 and expressed in at least three single cells were included, which left 20,262 genes across 2344 samples for clustering analysis. After initial clustering, *PAX6*, *NEUROD2*, *GAD1*, *PDGFRA*, *AQP4*, and *PTPRC* were used as markers to identify the major cell types in the brain: i.e., neural progenitor cells, excitatory neurons, interneurons, oligodendrocyte progenitor cells, astrocytes, and microglia, respectively.

### Coding sequence evolutionary analysis of *RBFOX1*

To analyze the evolution of the coding regions of *RBFOX1*, we obtained the human, chimpanzee, rhesus macaque, marmoset, mouse lemur, mouse, rat, cow, dog, and opossum coding sequences for this gene from Ensembl [48]. The coding sequences were aligned using Prank [73]. Gblocks v0.91b was used to remove poorly aligned regions in the resulting nucleotide sequence alignments [74]. We then used the modified branch-site model A from the PAML package v4.9 to test positive selection of *RBFOX1* in the human and primate lineages, respectively [75]. The null hypothesis of the branch test was that all lineages shared the same *dN*/*dS* ratio. The alternative hypothesis was that human or primate lineages had a different *dN*/*dS* ratio from other lineages, with w0, w1, and w2 representing codons under negative, null, and positive selection, respectively. The Chi-square test was used to calculate the *P* value P_adjust< 0.05 was considered as significant.

## Supplementary information

**Additional file 1: Supplementary Tables S1–S12.**

**Additional file 2: Supplementary Figure S1–S5.**

## Data Availability

The RNA-seq and microarray dataset of human analysed during the current study are available in the http://www.brainspan.org/static/download.html repository [24]. The microarray dataset of macaque analysed during the current study are available in the http://blueprintnhpatlas.org/static/download repository [25].

## Abbreviations

PCW: Postconception week
DNB: Dynamic network biomarker
NHPs: Non-human primates
RNA-seq: RNA-sequencing
HIP: Hippocampus
STR: Striatum
ACC: Anterior cingulate cortex
AMY: Amygdala
V1C: Primary visual cortex
DEGs: Differentially expressed genes
FC: Fold change
GW: Gestational week
WGCNA: Gene co-expression network analysis
MDC: Modular differential connectivity
GCM: Modules that show gain of connectivity
LCM: Modules that show loss of connectivity
NCM: Modules that show no change of connectivity
*RBFOX1*: RNA Binding Fox-1 Homolog 1
HACNSs: Human-accelerated conserved non-coding sequences
HARs: Human DNA sequence accelerated regions
TPM: Transcripts per million
