# Supplementary material for: Evolution and transition of expression trajectory during human brain development
Source: BMC Evol Biol. 2020 Jun 23;20:72. doi: 10.1186/s12862-020-01633-4 (PMC7310562; doi:10.1186/s12862-020-01633-4)
Supplement: Supplementary file 2 — Additional file 2: Supplementary Figure S1–S5. [file 12862_2020_1633_MOESM2_ESM.docx]

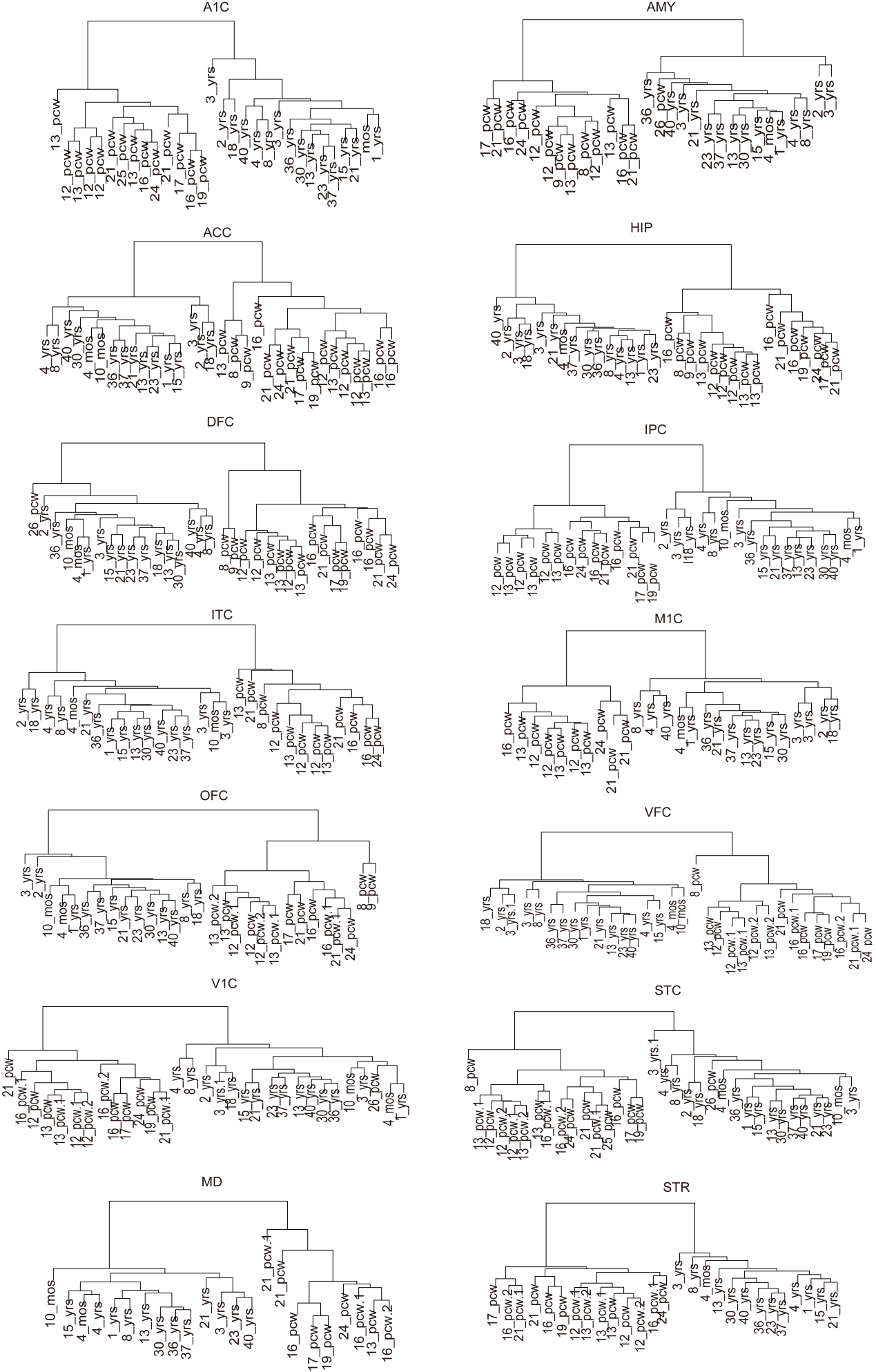


**Fig.S1** Clustering of genes expression in each tissue of human


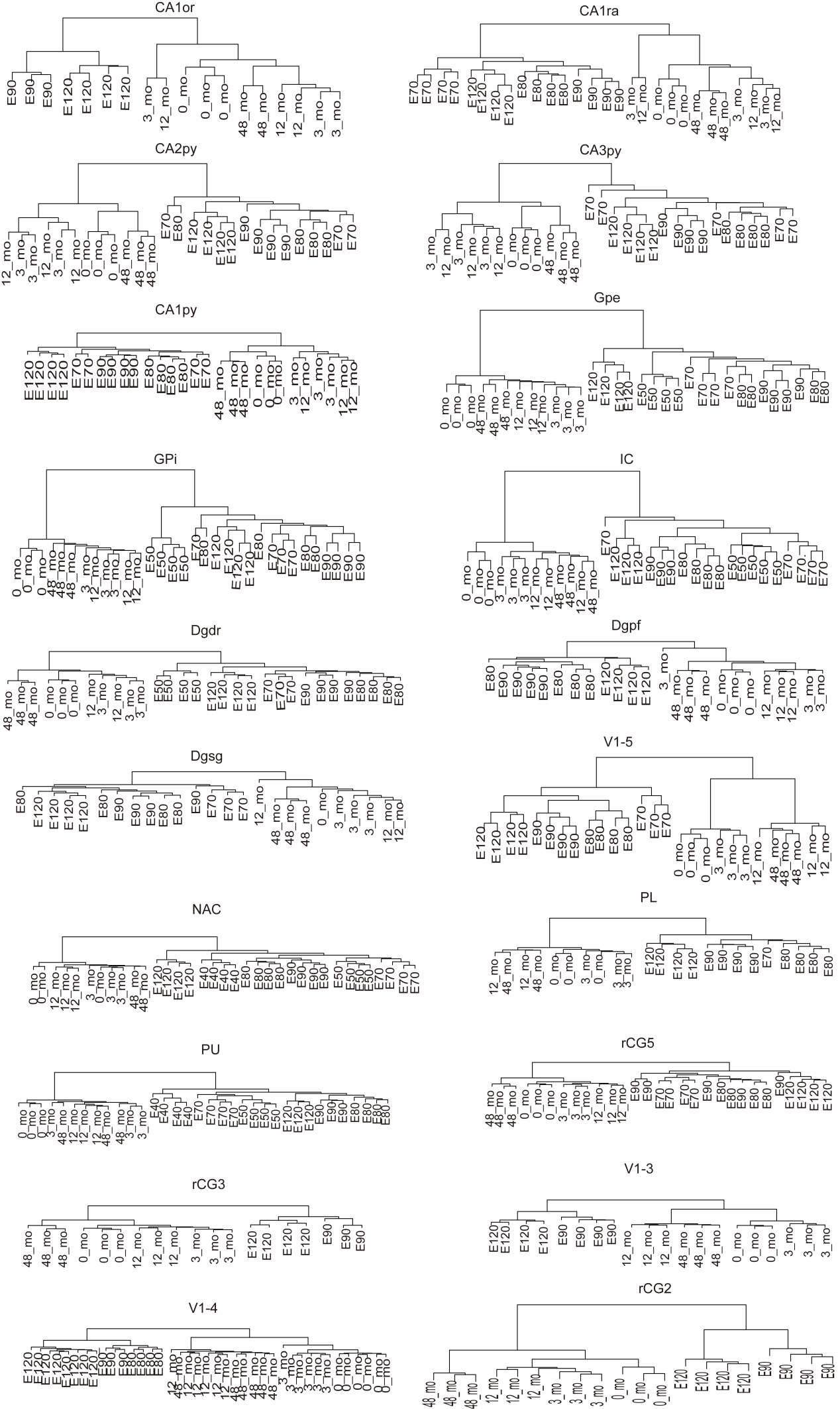


**Fig.S2,** Clustering of genes expression in each tissue of macaque


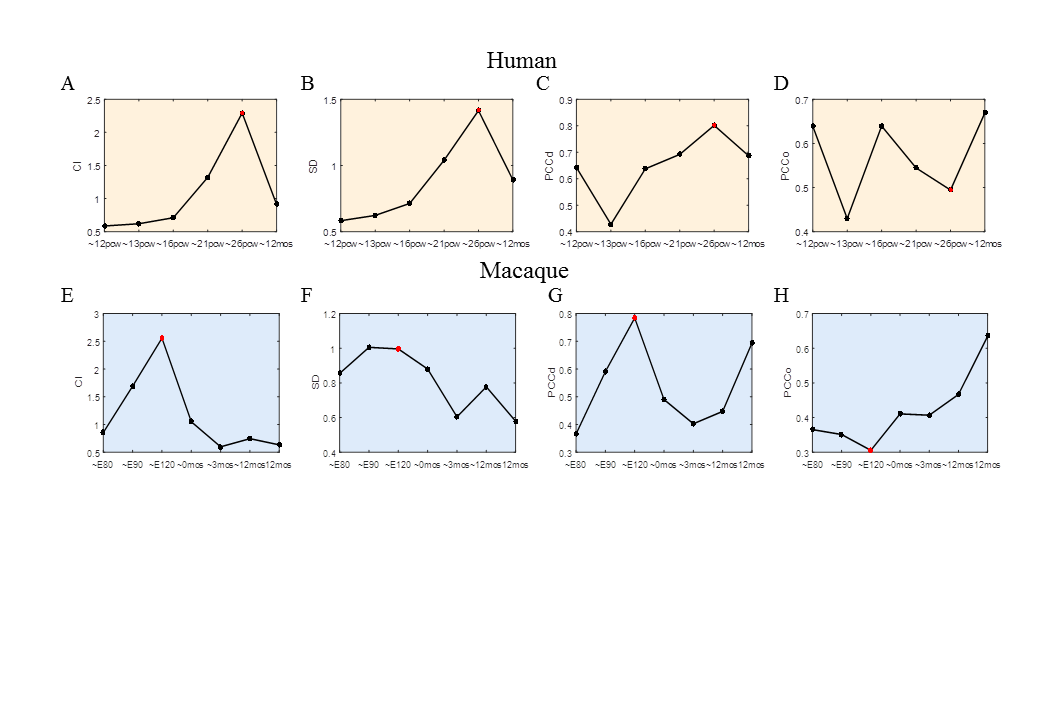


**Fig.S3. Detecting the tipping points during the brain development of human and macaque by DNB**.

Detecting the tipping points for two data sets, i.e. human (A, B, C, D) and macaque (E, F, G, H). The human data contain 17 samples (age range from 12pcw to 1 year old). The macaque data contain 24 samples (age range from E70 to 1 year old). Subfigures A and E represent the composite indices (see Materials and methods, CI in Eq.(1)), Subfigures B and F represent the mean SDs in the DNB of human and macaque (see Materials and methods, SD in Eq.(1)), Subfigures C and G represent PCCs in the DNB(see methods, PCCd in Eq.(1)), Subfigures D and H are the PCCs between the DNB and other molecules (see methods, PCCo in Eq.(1)).The results of the figure show the effectiveness of the DNB model by using two data sets, which identified that around 26pcw and around E120 are the tipping points of two species during the development.


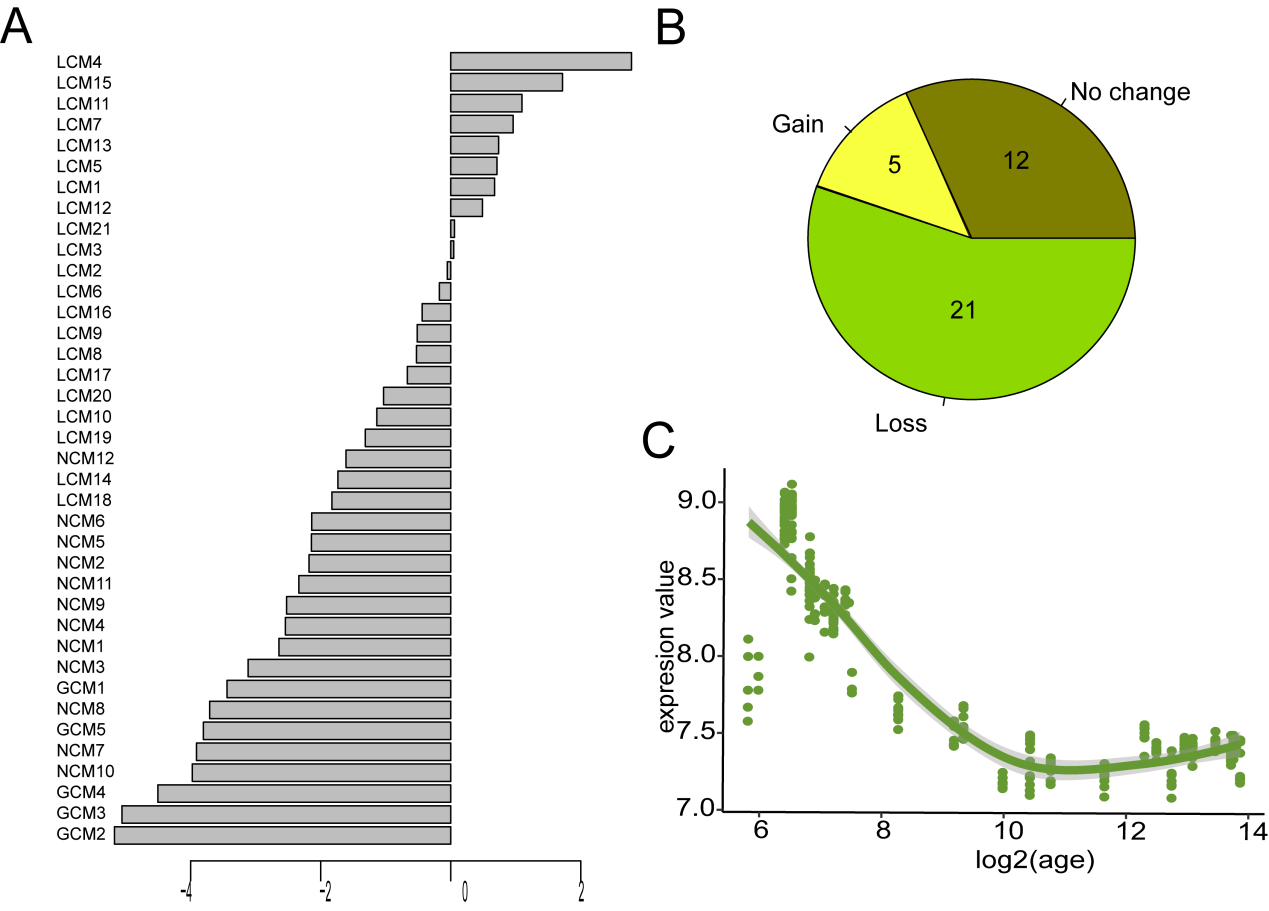


**Fig.S4.** Module differential connectivity analysis and expression trajectory of GCM1.

**(A)** In early stage, 5 modules showed gain of connectivity, 21 showed loss of connectivity, and remained 12 showed no change in connectivity.

**(B)**  Pie charts summarize module differential connectivity (MDC) analysis

**(C)** Expression trajectory of genes in GCM1


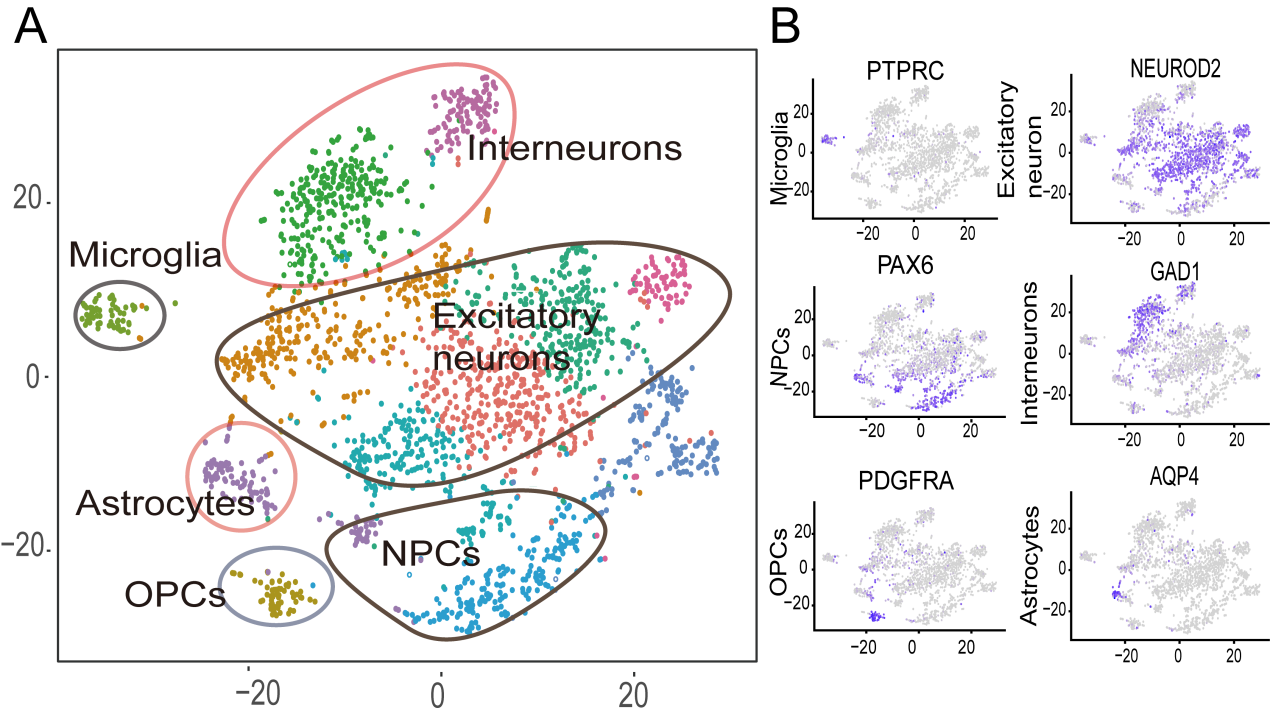


**Fig.S5.Single cell analysis from public data[**[**1**](#_ENREF_1)**].** Visualization of major classes of cells using *t*-SNE(left). Expression of known markers(right).

1. Zhong S, Zhang S, Fan X, Wu Q, Yan L, Dong J, Zhang H, Li L, Sun L, Pan N *et al*: **A single-cell RNA-seq survey of the developmental landscape of the human prefrontal cortex**. *Nature* 2018, **555**(7697):524-528.
